# Headache and Other Pain Syndromes in Multiple Sclerosis: A Narrative Review

**DOI:** 10.3390/life14010087

**Published:** 2024-01-05

**Authors:** Carmen Adella Sîrbu, Andreea Ruxandra Rotaru, Florina Anca Antochi, Andreea Plesa, Aida Mihaela Manole, Adina Maria Roceanu

**Affiliations:** 1 Clinical Neurosciences Department, “Carol Davila” University of Medicine and Pharmacy, 050474 Bucharest, Romania; 2 Department of Neurology, “Dr. Carol Davila” Central Military Emergency University Hospital, 134 Calea Plevnei, 010242 Bucharest, Romania; ruxandra_rot@yahoo.co.uk; 3 Academy of Romanian Scientists, 050045 Bucharest, Romania; 4 Neurology Department, University Emergency Hospital, 050098 Bucharest, Romania; amr2012mar@gmail.com; 5 Doctoral School, Faculty of Medicine, “Carol Davila” University of Medicine and Pharmacy, 050474 Bucharest, Romania; andreea_plesa28@yahoo.com (A.P.); aidamihaelamanole@gmail.com (A.M.M.)

**Keywords:** multiple sclerosis, migraine, tension-type headache, MRI, trigeminal autonomic cephalalgias, cluster headache, SUNA, SUNCT, pain syndromes, DMTs

## Abstract

Multiple sclerosis is a chronic and progressive neurological disease, with an important socio-economic burden. Over time, an increased incidence of headaches like migraines and tension headaches has been observed among these patients. Headaches have not been considered as multiple sclerosis-related symptoms, even representing a red flag for multiple sclerosis diagnosis. It is uncertain whether the headache–multiple sclerosis association could be explained by the presence of common triggers or a common physiopathological mechanism (involvement of tertiary B-cell follicles). An important differential diagnosis is between multiple sclerosis attacks and migraines with aura, which can also be associated with neurological deficits. Another important aspect is the occurrence or exacerbation of the cephalalgic syndrome after the initiation of therapy for multiple sclerosis (DMTs), or the improvement of headache after the initiation of certain DMT drugs. In addition to headaches, individuals diagnosed with multiple sclerosis often report experiencing diverse pain syndromes, contributing to an additional decline in their overall quality of life. These syndromes are frequently neglected, the focus being on slowing down the progression of neurological deficits. This review aims to evaluate the characteristics of multiple-sclerosis-related headaches (frequency, possible correlation with attacks, and disease-modifying therapies) and the key distinctions in imaging characteristics between demyelinating lesions in multiple sclerosis and those observed in cases of primary headaches.

## 1. Introduction

Multiple sclerosis (MS) is a chronic disease involving the central nervous system, which is based on inflammation, demyelination, and neurodegeneration processes. It affects around 2.2 million people worldwide, mostly young adults [1]. In most cases, the disease initially develops in attacks with variable severity and duration, with typical remission after a few weeks. Along with the evolution of the disease, the degree of disability increases, and the symptomatology is no longer intermittent.

In addition to motor and sensory deficits, this disease can also be associated with painful manifestations such as headaches, temporomandibular joint pain, Lhermitte’s sign, several types of neuralgia (occipital neuralgia, trigeminal neuralgia), muscle spasms, and restless legs syndrome, all this leading to a supplementary decrease in the patient′s quality of life [2].

Considering the increased incidence of headaches in MS, the physician must be aware of the diagnosis and management of common headache types. These patients frequently have primary headache disorders, with migraines taking the lead, followed by tension-type headaches and then cluster headaches. Depending on the case, secondary causes should be investigated and excluded. Even if disease-modifying drugs (DMTs) are the focus of MS treatment, they can lead to the worsening of pre-existing headaches or their appearance as a side effect. Therefore, proper knowledge of the therapeutic agents and their effects can help to treat headaches and improve compliance.

## 2. Materials and Methods

We delved into the exploration of syndromes and their connection to multiple sclerosis (MS) through a comprehensive review of the existing literature. Our search encompassed renowned databases, including PubMed, Scopus, NCBI, and relevant medical journals. Utilizing specific search terms like “migraine”, “multiple sclerosis”, “DMT” (Disease Modifying Therapy), “secondary headache”, and “pain and MS”, we meticulously directed our exploration. A conscious effort was made to prioritize recent studies, articles, and reviews.

In the course of our study, we systematically considered relevant literature published between 1 January 2005, and 17 May 2023. We applied exclusion criteria, filtering out publications deemed irrelevant in terms of type, those lacking a complete English text, duplicate studies, and materials with insufficient methodological clarity. An author extracted the data using a standardized protocol, and another author conducted a thorough check to ensure accuracy and reliability.

During the synthesis and analysis of the collected data, our aim was to construct a coherent narrative that unveils the current state of knowledge regarding the intricate relationship between headaches, other pain syndromes, and MS.

While narrative reviews play a crucial role in synthesizing existing literature, they present certain considerations. These include subjectivity, incomplete coverage, limited reproducibility, and the evolving nature of knowledge. We acknowledged these limitations in formulating conclusions and discussing potential implications for future research.

## 3. Types of Headaches in Multiple Sclerosis Patients

The main types of headache (tension-type headaches, migraines, and cluster headaches) are also present in the case of multiple sclerosis patients, but with some percentage differences, depending on the studied population. Migraines and MS share some common background factors, including gender (with a female predominance), hormonal status (with premenstrual or postmenopausal variations), and psychological feature periods accompanied by anxiety, depression, stress, the diagnosis itself leading in many cases to this type of manifestation) [3]. It was also observed that both migraine frequency and MS activity were reduced during pregnancy. Neuroinflammation is the process that seems to play a key role in the physiopathogenesis of both diseases, with at least one common downstream pathway.

Female sex is a relevant risk factor for migraines, not for tension-type headaches. No link was found between age and one of the types of primary headaches, but it seems that the elderly associate migraines with aura more often [4]. Previous studies have shown a different onset time depending on the type of primary headache; in a significant number of cases the migraine attacks started before the MS diagnosis, while tension-type headaches seemed to begin after MS onset [4]. In terms of the clinical presentation of demyelinating diseases, research has indicated a higher occurrence of migraines in individuals diagnosed with relapsing–remitting forms of multiple sclerosis, whereas the tension-type headache had a higher frequency in patients with chronic progressive MS [5,6].

When considering the types of headaches experienced by individuals with multiple sclerosis, it is notable that migraines rank as the most prevalent, with a prevalence of 31% [7], followed by tension-type headaches. Cluster-type headaches have been reported in a few cases. There have been rare instances of cluster-type headaches reported among these patients. The infrequency of cluster-type headaches in this context could be attributed to their low prevalence in the general population [8,9]. Migraines associated with multiple sclerosis represent the subject of many studies. Initially, they were regarded as an exclusion criterion for MS [10] and continue to be considered a red flag in clinical assessments. The similarity in clinical presentation can frequently lead to difficulties in distinguishing between a multiple sclerosis relapse and a migraine aura.

In 1952, Compston made the first assumption about a potential connection between migraine and multiple sclerosis. It remains uncertain whether these two diseases are causally linked or if they are only comorbid conditions. It has been shown that brain stem and upper cervical demyelinating lesions (situated in the C2 dorsal horn) are associated with headaches in MS [11,12]. On the other hand, patients reported the occurrence or exacerbation of the cephalalgic syndrome after the initiation of therapy for multiple sclerosis (disease-modifying drugs).

Elderly patients, with longer disease evolution and a lower SDMT score, complain more often of migraines with aura [13]. MS patients who associate migraine headaches seem to have typical migraine characteristics but with a more important severity regarding the intensity of pain and a high frequency of headache attacks [13]. The most frequent headache triggers described in the studies were fatigue and stress [8]. It has also been shown that stress precipitates MS exacerbations. Other migraine triggers in the MS population were weather change and sleepless nights [14]. All of these can be improved through a balanced lifestyle and an individualized work schedule.

Several hypotheses regarding the association between multiple sclerosis and migraine have been proposed: disrupting the pathways which are involved in the pathogenesis of migraines by the inflammatory demyelinating MS lesions can induce headache episodes [15]; there is a connection between headache exacerbation and malfunction of the serotoninergic system caused by demyelinating lesions [16]; demyelination with cortical localization has been shown to speed up cortical spreading depression, this being a key mechanism involved in migraine pathophysiology [17].

## 4. Is Headache in Multiple Sclerosis Primary or Secondary?

Primary headaches in the context of multiple sclerosis refer to headache disorders that are not directly attributable to the underlying neurological manifestations of MS or other pathologic processes, disease, or traumatic injury. The most common types of primary headaches according to the ICHD-3 revised headache classification are migraine, tension-type headaches, trigeminal autonomic cephalalgias, and other primary headache disorders. These headaches are characterized by their own distinct clinical features, triggers, and patterns, and they are not directly correlated with the progression or activity of MS.

Secondary headaches within the context of multiple sclerosis (MS) are those that emerge directly as a result or consequence of the neurological features or complications associated with MS. These headaches are intricately linked to underlying pathological processes, including demyelination, inflammation, or other structural changes within the central nervous system. They may vary in presentation and may include features such as the exacerbation of pre-existing primary headaches, headaches associated with MS-related sensory disturbances, or headaches triggered by MS-related complications, such as medication side effects or comorbid conditions. Identifying and addressing the underlying MS-related factors is crucial in managing secondary headaches in this context.

The overall prevalence of primary headaches among patients with MS is considerably high, being estimated at approximately 56% [18].

Patients receiving disease-modifying drugs complained more frequently of headaches at some point during their illness compared to patients undergoing other forms of therapy [10]. Based on a recent study, over half of the patients treated with disease-modifying therapies (DMTs) exhibited primary headaches [13].

**Figure 1:**
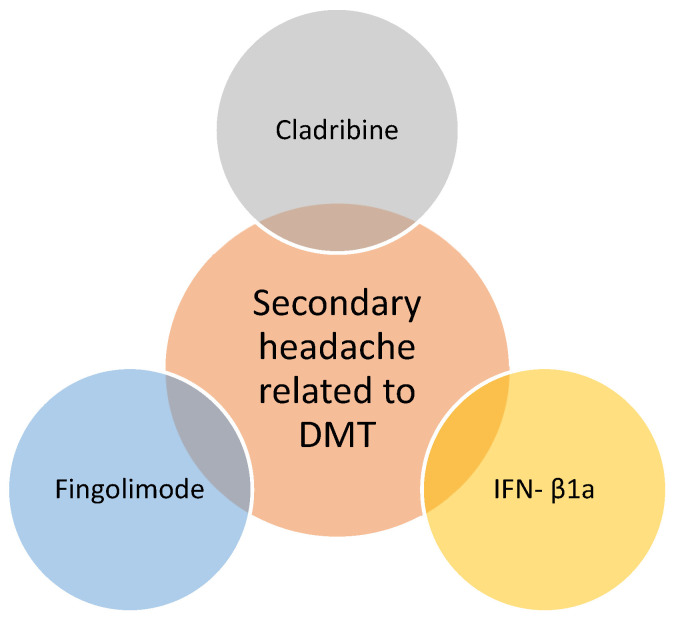
DMT related to headache in multiple sclerosis.

**Figure 2:**
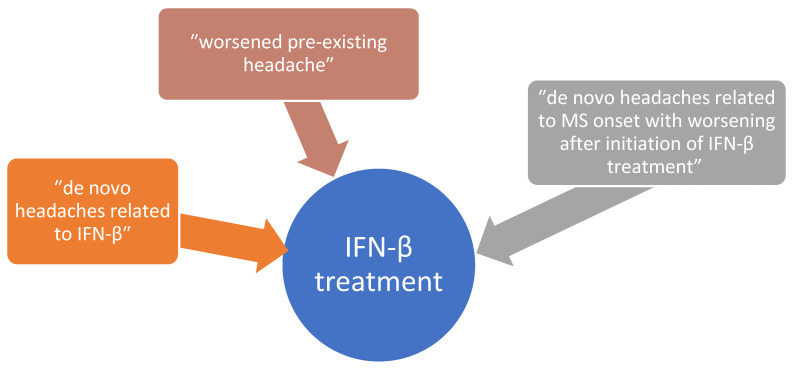
Types of headaches in SM patients treated with IFN-β.

Headaches have been observed as side effects since the appearance of the first types of DMTs (Figure 1). It has been shown that interferon-beta as a disease-modifying therapy is associated in many cases with exacerbation of preexisting migraines and worsening headaches, including increased frequency, increased intensity, and increased duration. More different types of headaches after IFN-β treatment initiation are described in studies, these being listed in the figure below [19] (Figure 2). The same study notes that patients complain of de novo headaches even from the first 4 weeks after starting the IFN-β treatment and concluded that both the exacerbation of pre-existing headaches and the new onset of headaches can be classified as adverse drug effects.

The PRISMS trial described an association between patients treated with IFN-β1a and headaches in 71% of participants. Similar percentages were confirmed by other studies.

Other studies revealed cases of patients previously diagnosed with headaches whose characteristics changed after receiving IFN-β treatment which led to reconsideration of the initial diagnosis from tension-type cephalalgic syndrome to migraines without aura [20].

B-cell targeted therapies (Ocrelizumab, Rituximab, Ofatumumab, Ublituximab) were initially believed to be a potential cause of headaches as a side effect. However, recent research has contradicted this assumption. A recent study demonstrated that B-cell targeted therapies for multiple sclerosis (MS) are not significantly linked to an increased incidence of headaches as an adverse effect. Interestingly, the study revealed a statistically significant association between cladribine and a higher occurrence of headaches. Further studies are required to elucidate the mechanism of headaches associated with cladribine treatment, aiming to identify an effective strategy for prevention [21]. Natalizumab does not seem to aggravate a comorbid migraine, but some cases with headaches as infusion-related short-term reactions have been related [22].

A study involving a limited number of patients indicated that commencing therapy with fingolimod led to the onset of headaches within the initial week, and these headaches persisted for a minimum of three months [23]. The patients in treatment with other DMTs such as teriflunomide, dimethyl fumarate, and glatiramer acetate did not complain of headaches as a secondary reaction [24,25].

Overuse of pain relievers for other pain symptoms associated with MS (such as neuropathic pain or pain caused by spasticity) can also lead to headaches.

In patients presenting with worsening or new paroxysmal headaches, it is necessary to exclude secondary causes.

## 5. Imaging Differences between the Demyelinating Lesions in Multiple Sclerosis and Those That Appear in the Case of Migraines

Brain white matter lesions appear as non-specific small, round, and non-expansile focal lesions situated subcortical, infratentorial, periventricular, or deep in the white matter. These lesions can be present both in healthy adults and in patients diagnosed with migraine or vascular disease. These are best observed on T2 and fluid-attenuated inversion recovery (FLAIR) IRM sequences, in many cases being an incidental discovery. It is seldom feasible to distinguish their origin—whether these headaches are a result of demyelination or a comorbid condition [17]. The similar radiological appearance of conventional MRI can be explained by overlapping pathophysiological mechanisms. Sometimes the doctor can follow a diagnostic plan depending on the disposition of the white matter lesions, these being discussed below.

Imaging analyses suggest that migraines appear to be an independent risk factor for specific locations of deep white matter lesions, such as those situated supratentorial, infratentorial, and in some cases, silent infarcts in the posterior circulation territory (especially in the case of migraineurs with aura) [26].

It should be noted that white matter lesions in migraine patients seem to occur earlier in life [27] and these are present in up to 10% of pediatric migraineurs [28].

The differentiation of migraine and MS-related lesions is important because the appearance of new hyperintense lesions can influence the therapeutic decision. Table 1 shows the main imagistic features of white matter lesions in MS compared to migraines.

To support the spatial dissemination criteria of the multiple sclerosis diagnostic algorithm, the lesions must have a certain location—at least one typical demyelinating lesion in two or more specific regions, periventricular—very close to the ventricles without intervening white matter, juxtacortical and cortical, infratentorial, or spinal cord [26]. The typical shape of multiple sclerosis lesions is round to ovoid and the diameter can vary, ranging from a few millimeters to more than one or two centimeters. To meet the diagnostic criteria, the long axis of these lesions should measure at least 3 mm. A common IRM characteristic of MS and lacunar infarcts is that multiple sclerosis lesions appear bright and hyperintense on T2 and FLAIR sequences and when these become chronic they appear in a low T1 signal [17].

In the case of migraines, the lesions can be found in deep white matter from all four lobes, as well as in the callosal commissure, periventricular, or juxtacortical. Migraine-related lesions have the following characteristics: a round or slightly elongated shape with a diameter generally smaller than 3 mm, punctate, focal, and non-confluent [29]. Periventricular, basal ganglia infratentoria, brainstem, or other posterior fossa structure lesions are uncommon [30].

For the differential diagnosis of lesions (migraine-related, lacunar infarcts, MS-related, or with microangiopathic substrate), it is important to perform T1-weighted sequences, where the lesions associated with migraine are not as hypointense [30]. Encephalomalacia is the process that gives the T1 hypointense appearance, so the absence of a low signal on T1 in migraines shows that this pathology is based on a different mechanism.

The large number of white matter lesions in the elderly population makes it difficult to differentiate the advanced-age-related lesions from those that are migraine-related [31]. In young patients, it is necessary to exclude a wide range of vascular disorders to conclude that white matter lesions are migraine-related.

Periventricular white matter lesions, often discovered incidentally in the elderly population, predispose these patients to a higher risk of dementia, cognitive decline, and stroke. Studies have shown no link between white matter lesions in migraineurs and the occurrence of cognitive changes [32].

White matter hyperintensities are prevalent in a significant proportion of female migraine patients, with more than one-third affected, with a higher frequency in the case of migraines with aura [33].

A study showed that the accumulation of disability during attacks has an imaging resonance by a proportional increase in the number of white matter lesions [34].

It was observed that certain white matter lesions are temporary and may resolve over time, being associated with migraine attacks. This reversible character is related to dysfunctions of the hematoencephalic barrier, which lead to an enhanced permeability of meningeal microvasculature and vasogenic edema [35].

Another study showed that certain locations for demyelinating lesions might be some of the factors responsible for the coexistence of migraines in patients with MS. These locations are represented by the substantia nigra, red nucleus, and periaqueductal grey matter [36].

Several recent studies have used the identification of central vessel signs using inversion recovery sequence (FLAIR) on 3T MRI for the differentiation of MS lesions from migraine ones [37], but this method needs to be analyzed further in other studies.

## 6. Other Pain Syndromes in MS Patients

Pain is a disabling medical condition with a significant adverse effect on the individual’s quality of life. Neuropathic pain is a symptom that is frequently reported by patients with multiple sclerosis, with a fairly variable prevalence between 25% and 90%, and it is a chronic pain, associated with depression and significant MS-related disability [38]. Patients usually complain of this type of pain from the early stages of the disease. The most common types of cranial neuralgias, including occipital neuralgia, trigeminal, and glossopharyngeal neuralgias, can appear in the case of MS patients.

The pathophysiological mechanisms underlying trigeminal neuralgia encompass several intricate factors. One significant aspect involves the compression exerted on the trigeminal nerve root. Additionally, conditions characterized by primary demyelination play a contributory role in the development of trigeminal neuralgia. Furthermore, the sensitization and dysfunction of central circuits associated with pain processing contribute to the complexity of this neurological disorder. These multifaceted elements collectively contribute to the manifestation and progression of trigeminal neuralgia [39]. Neuralgia secondary to MS has common characteristics with the classic one, it more often affects women and the right side of the face, but in a percentage of 18% of MS patients, the neuralgia is located bilaterally [40]. Also, it seems to appear at a younger age. To diagnose secondary trigeminal neuralgia, it is necessary to observe the presence of a structural abnormality that involves the path of the trigeminal nerve other than vascular compression. These may include abnormalities or tumors located at the level of the skull base and demyelinated plaques which are multiple-sclerosis-related, with a preponderance of the latter. MS patients have a significantly higher risk of developing trigeminal neuralgia [41]. MRI is the preferred diagnostic method to show evidence of trigeminal pathway damage, generally, with plaques located in the ventrolateral pons. Neurophysiological investigations can also be performed to support the diagnosis: trigeminal reflex testing having a high sensitivity and specificity [42]. In patients experiencing neuralgia secondary to multiple sclerosis, a substantial 89% exhibited abnormal results in the trigeminal reflex test. In contrast, individuals with idiopathic neuralgia displayed abnormal test results in only 3% of cases [43]. Trigeminal neuralgia in most MS patients has an intermittent evolution, with periods of remission. Hypotheses have been issued according to which the cause of this type of neuralgia is represented by a reduction in neuronal excitability and partial remyelination, without evidence to support them [39]. In several cases, imaging investigations followed by surgical treatment suggest a double-crash mechanism (neurovascular compression in concern with pontine plaque) [44]. Although classical trigeminal neuralgia and trigeminal neuralgia secondary to MS present similar characteristics, MS patients more frequently reported bilateral pain, with an estimated percentage of 18% [45,46].

A study conducted by the MS Treatment and Research Center of Minneapolis which included a small batch of patients (71 persons) suggests that fampridine can cause a reactivation of neuropathic pain due to trigeminal neuralgia [47].

Occipital neuralgia is characterized by paroxysmal intense pain that feels like a sharp, jabbing, electric shock in the distribution of one of the three major occipital nerves. It can also be associated with a decreased sensation or dysesthesia. Occipital neuralgia is in most cases idiopathic. The other etiologies include neurosyphilis, C1-C2 arthrosis, corticomedullary dural arteriovenous fistula, vascular compressions, upper cervical cavernous angioma, and MS [48]. A recent study concluded that occipital neuralgia may be a symptom of MS relapse or the initial symptom of MS [49]. Considering that it is not a common pathology (incidence and prevalence are unknown), suspicion of MS in cases with occipital neuralgia could reduce the delay in diagnosis of demyelinating disease and decrease disability by enabling early treatment. In some cases, occipital pain with intense and paroxysmal onset can signal a relapse of MS, and these patients need a cervical MRI with gadolinium for high diagnostic accuracy [50].

SUNCT (short-lasting unilateral neuralgiform headache with conjunctival injection and tearing) is a part of the group of trigeminal autonomic cephalalgias and is a relatively new nosological entity [51]. It represents a rare condition which is characterized by brief, unilateral, and painful attacks typically located in the orbital or temporal area and associated ipsilateral autonomic manifestations [52]. Although many cases reveal no apparent structural causes in imaging studies, SUNCT has been reported secondary to structural lesions in the posterior fossa. Short-lasting unilateral headache with cranial autonomic symptoms (SUNA) has a similar definition to SUNCT; this could involve any of the autonomic symptoms. In the existing literature, secondary conditions associated with these abnormalities encompass a limited number of cases involving multiple sclerosis patients [53]. Additionally, other examples documented in the literature include cortical dysplasia and patients with vertebral artery dissection. In patients with multiple sclerosis who present lesions of the posterior fossa, these two entities must also be taken into account.

Another paroxysmal pain syndrome in MS patients is Lhermitte’s sign, described as a painful, electric-current-like sensation in the spine when the patient flexes the neck. In 95% of the patients presenting this sign, cervical demyelinating lesions were detected during MRI examination [54].

Chronic pain, both nociceptive and neuropathic, is a common symptom that patients with multiple sclerosis often complain about [40]. It can appear at some time during disease, in a percentage of 50–75% of MS patients [38]. Neuropathic pain is most often chronic (duration over 12 weeks), present from the early stages of the disease, and in many cases it requires treatment. The main types of chronic neuropathic pain conditions have been described above. It was noted that patients with the primary progressive form of the disease complain more often of dysesthesia [45].

## 7. Management of Pain in MS Patients

Symptomatic treatments are important in helping individuals to fulfil their personal, social, and occupational roles and improve their quality of life, for as long as possible. Unfortunately, any conventional pain medication offers complete pain relief, but even incomplete relief is important in the case of these patients. A temporary relief of pain can be obtained by taking antidepressants and anticonvulsants. Trigeminal neuralgia secondary to MS is treated in first-line therapy with sodium-channel blockers such as carbamazepine or oxcarbazepine [44]. Other therapeutic options include phenytoin, lamotrigine, and gabapentin [55]. The residual pains that persist between paroxysms are often resistant to sodium-channel blockers [56]. Medication-resistant patients can respond to low-dose aripiprazole. In selected cases, baclofen and misoprostol can be used to relieve neuralgia. The last drug can help in nerve stabilization by decreasing the inflammatory activity in the plaques [57]. Tramadol and opioid analgesics can be used as second-line therapies, for limited periods [58]. If pharmacological treatment fails, neurosurgical procedures such as rhizotomy, microvascular decompression in the posterior fossa, Gasserian ganglion percutaneous techniques, or gamma knife can be considered. Surgical methods seem less effective than in idiopathic trigeminal neuralgia.

Regarding the treatment of migraines, CGRP-specific therapies require additional studies to establish the safety of their association with DMTs.

Amitriptyline may be prescribed for patients complaining of chronic dysaesthetic pain. Regarding Lhermitte′s sign, treatment with oxcarbazepin, carbamazepine, and gabapentin can be useful in some patients [59].

Pain related to spasticity often improves with adequate physiotherapy. Drug treatment includes antispastic agents such as baclofen or tizanidine and in patients with phasic spasticity, gabapentin or levetiracetam are administered. In patients with severe spasticity, botulinum toxin injections or intrathecal baclofen merit consideration [60].

An adjuvant treatment that can be used in MS patients to improve sensory symptoms is transcranial magnetic stimulation (TMS). This method does not seem to interact with DMTs [61]. In addition to that, TMS can also alleviate spasticity, gait disturbance, and numbness, with patients also noticing positive effects on fatigue [62].

Another treatment option is transcutaneous electrical nerve stimulation, a therapy used effectively to improve post-stroke spasticity. A study that analyzed its use in the case of multiple sclerosis patients suggested a low effect in terms of spasticity, but it seems to be a good solution for relieving muscle spasms and pain [63].

Depression is an underdiagnosed and undertreated condition both in the general population but especially in the case of patients with neurological conditions, being associated in many cases with receiving a diagnosis with an important psychosocial impact. Formal screening tools for depression are deemed crucial in studies involving patients with chronic or refractory headaches, with a particular focus on those experiencing migraines [64]. Some of the most-used tests in studies that include patients with migraines are the Hospital Anxiety and Depression Scale (HADS) and PHQ-9 [65]. Regarding the association of depression with MS, studies have shown percentages between 20% and 40% [66,67]. Screening for depressive symptoms is crucial for patients with MS and headaches.

In terms of psychological counselling, mindfulness interventions and cognitive behavioral therapy are beneficial for these patients, leading to stress reduction and the improvement of depressive symptoms. Improvements were also noted in terms of fatigue, pain, reduction in the intensity of symptoms, anxiety, and tolerating uncertainty.

Some lifestyle changes such as limiting alcohol and caffeine, meditation, a regular sleep schedule, a balanced diet, and maintaining an active social life can lead to a reduction in painful symptoms.

## 8. Discussion

Already in 1952, the first hypothesis was issued that there is a connection between migraines and MS. Migraines had the highest incidence in MS patients over time, followed by tension-type headaches. The elevated prevalence of both migraines and multiple sclerosis among patients might be attributed to common risk factors (genetic background, environmental exposure). This relationship needs additional studies. Patients who were diagnosed at young ages and with the relapsing–remitting MS form may complain of headaches since the onset of the disease.

Secondary headaches can be correlated with the initiation of disease-modifying therapies, in particular, IFN-β, and can include both changes in pre-existing headache characteristics and the appearance of “de novo headaches”. These symptoms, coupled with flu-like symptoms, can significantly impair their quality of life and adherence to therapy. Being a frequent pathology in MS patients, studies are needed to analyze the influence of other DMTs on headaches.

In the end, is the headache a secondary type, being considered the manifestation of MS, or is it a different entity that is exacerbated during relapses of the demyelinating disease? While a definitive answer remains elusive, recent research indicates that the coexistence of migraines and multiple sclerosis (MS) is likely linked to shared genetic variants. Four key loci within the major histocompatibility complex have been identified in both conditions [68]. Also, the subjects with both diseases were generally smokers. The same study suggests that migraines are rather a consequence of MS.

The imagistic differentiation of migraines and MS-related lesions is important because the appearance of new hyperintense lesions can influence the therapeutic decision. Even if the imaging appearance of white matter lesions is similar, in the case of younger patients we can be guided toward a migraine or MS etiology by following their shape, size, and location, excluding a wide range of vascular disorders.

The most common types of cranial neuralgias, including occipital neuralgia, trigeminal, and glossopharyngeal neuralgias, can appear in the case of MS patients. MS patients have a significantly higher risk of developing secondary trigeminal neuralgia.

A cervical MRI with gadolinium is indicated in the case of posterior headaches to differentiate secondary occipital neuralgia or an MS relapse.

Questionnaires about headaches, as well as their investigation, should be included in the neurological examination of all MS patients.

Both migraines with aura and occipital neuralgia can hide an MS relapse, requiring additional investigations.

Regarding the social impact of these patients, it has been observed that they have low self-esteem, low levels of marital satisfaction, an increased risk of suicide, and early retirement from employment.

The quality of life of MS patients can be improved through an early diagnosis and adequate therapeutic management of these pain syndromes.

## 9. Conclusions

In summary, headache and other pain syndromes are much more frequent in MS patients than in the general population. In the case of migraine, which is frequently associated with white matter lesions, an extended etiological screening is essential until a definite diagnosis is established. In patients diagnosed with MS and migraine, the most important differential diagnosis is between a migraine episode with aura and an MS relapse.

Pain management, both pharmacologically and adjunctive including psychotherapy, is important in increasing the quality of life in these patients.

## Figures and Tables

**Table 1:** Imagistic features of white matter lesions in MS compared to migraine.

White Matter Lesions	Location	Shape	Size	T2, FLAIR	T1
Multiple sclerosis	periventricular, juxtacortical, cortical, infratentorial, spinal cord, perivenular distribution, orientation perpendicular to ventricular walls	round to ovoid	at least 3 millimeters to more than 1–2 cm	bright and hyperintense, central vessel signs *	low T1 signal
Migraine	deep white matter (lobes, callosal commissure, periventricular, or juxtacortical, centrum semiovale)	round or slightly elongated	smaller than 3 mm, punctate, focal, and non-confluent	bright and hyperintense	not as hypointense

* the central vessel sign can only be seen on 3 tesla MRI.

## Data Availability

No new data were created or analyzed in this study. Data sharing is not applicable to this article.
